# TGF-β- and lipopolysaccharide-induced upregulation of circular RNA PWWP2A promotes hepatic fibrosis via sponging miR-203 and miR-223

**DOI:** 10.18632/aging.102405

**Published:** 2019-11-13

**Authors:** Wentao Liu, Ruo Feng, Xingxing Li, Dingyang Li, Wenlong Zhai

**Affiliations:** 1 Department of Hepatobiliary and Pancreatic Surgery, The First Affiliated Hospital of Zhengzhou University, Zhengzhou 450000, China; 2 Department of Histology and Embryology, College of Basic Medical Sciences, Zhengzhou University, Zhengzhou 450000, China; 3 Department of Cardiac Surgery, The First Affiliated Hospital of Zhengzhou University, Zhengzhou 450000, China

**Keywords:** hepatic fibrosis, circ-PWWP2A, LPS, TGF-β, ceRNA

## Abstract

Both transforming growth factor-beta (TGF-β) and lipopolysaccharide (LPS) can activate hepatic stellate cells (HSCs), thus increasing expressions of alpha smooth muscle actin (α-SMA) and type I collagen alpha 1 (Col1α1) and promoting liver fibrosis. However, whether TGF-β and LPS have a common downstream reactor remains unclear. Recently, a strong relationship of circular RNAs (circRNAs) and fibrogenesis has been elucidated. In this study, we compared the expressions of several circRNAs in TGF-β- and LPS-activated HSCs, and found that circ-PWWP2A was upregulated in both TGF-β- and LPS-activated HSCs and in mouse fibrotic liver tissues. Meanwhile, circ-PWWP2A was positively correlated with HSC activation and proliferation. Two microRNAs, miR-203 and miR-223, were identified to be the downstream targets of circ-PWWP2A using luciferase reporter assay and pull-down interaction assay. Circ-PWWP2A was suggested to promote HSC activation and proliferation via sponging miR-203 and miR-223, and subsequently increasing Fstl1 and TLR4, respectively. Furthermore, downregulating circ-PWWP2A was indicated to alleviate hepatic fibrosis in vivo. In conclusion, our findings indicated that circ-PWWP2A is the common downstream reactor of TGF-β and LPS in HSC activation, and that circ-PWWP2A plays a critical role in hepatic fibrogenesis via sponging miR-203 and miR-223.

## INTRODUCTION

Hepatic fibrosis is the result of liver injury and repair, causing type I collagen-based collagen deposition in extracellular matrix (ECM) [1]. As a common pathological change of various chronic liver diseases, the uncontrolled hepatic fibrosis finally progresses to hepatic cirrhosis and leads to irreversible liver damage [2]. Hepatic stellate cells (HSCs) are the major participants of hepatic fibrosis as the activated HSCs promote collagen deposition in ECM by producing alpha smooth muscle actin (α-SMA) and type I collagen alpha 1 (Col1α1), both of which are critical markers of HSC activation [3]. Therefore, the inhibition of HSC activation is becoming a promising therapeutic strategy of hepatic fibrosis in recent years [4].

HSC activation is driven by a panoply of signals, including fibrogenic cytokines and innate immune signals [5]. Transforming growth factor-beta (TGF-β) is a critical fibrogenic cytokine, and can be secreted by several cell populations in the liver [6]. By binding with the surface TGF-β receptor, TGF-β activates the transcription factors Smad2 and Smad3, promoting transcriptions of type I and type III collagen, thus transforming HSCs into myofibroblast-like cells and accelerating hepatic fibrosis [7, 8]. Innate immune signals refer to the bacterial-derived metabolites, such as lipopolysaccharide, which is a component of the outer wall of the gram-negative bacteria, and maintains a low concentration in liver [9]. During acute and chronic hepatic pathogenesis, the translocation of LPS is elevated due to the increased intestinal epithelial permeability, activating multiple signaling pathways by binding with Toll-like receptor 4 (TLR4), inducing secretion of several cytokines and chemokines that help collagen accumulation in ECM [10]. Thus, TGF-β and LPS play an important role in HSC activation, and the blocking of the common downstream reactor of both TGF-β and LPS brings the possibility of inhibiting HSC activation. However, such downstream reactor has not been elucidated.

In recent years, a strong relationship of circular RNAs (circRNAs) and fibrogenesis has been elucidated. CircRNAs, a novel type of endogenous non-coding RNAs, are characterized by covalently closed loop structures with neither 5’ to 3’ polarity nor a polyadenylated tail [11, 12]. CircRNAs often have a high stability and are less susceptible to degradation as the loop structure is resistant to RNase R and RNA exonuclease [13]. The conserved and tissue-specific expression of circRNAs is reported and the strong relationship of circRNAs and various types of diseases, including type 2 diabetes, cancers, and Alzheimer’s disease, has been elucidated [14–16]. Recently, literatures reported that circRNAs exert their regulatory functions via acting as a competing endogenous RNA (ceRNA) to sponge microRNAs (miRNAs), another type of endogenous non-coding RNAs [17]. For example, circRNA CDR1as is reported to promote silica-induced pulmonary fibrosis by sponging miR-7 [18]. Chen and his colleagues found several circRNAs are dysregulated in irradiation-induced HSCs [19]. However, the effect of circRNAs in TGF-β- and LPS-induced HSCs has been largely unknown.

In the current study, the expression of several circRNAs was compared in TGF-β- or LPS-induced HSCs, and the circRNA PWWP2A (circ-PWWP2A) was markedly upregulated in both TGF-β- and LPS-induced HSCs. Meanwhile, circ-PWWP2A was demonstrated to promote the activation and proliferation of HSCs via sponging miR-203 and miR-223, and subsequently increasing Fstl1 and TLR4, respectively.

## RESULTS

### Circ-PWWP2A is upregulated in TGF-β- or LPS-activated HSCs and fibrotic liver tissues

**Figure 1:**
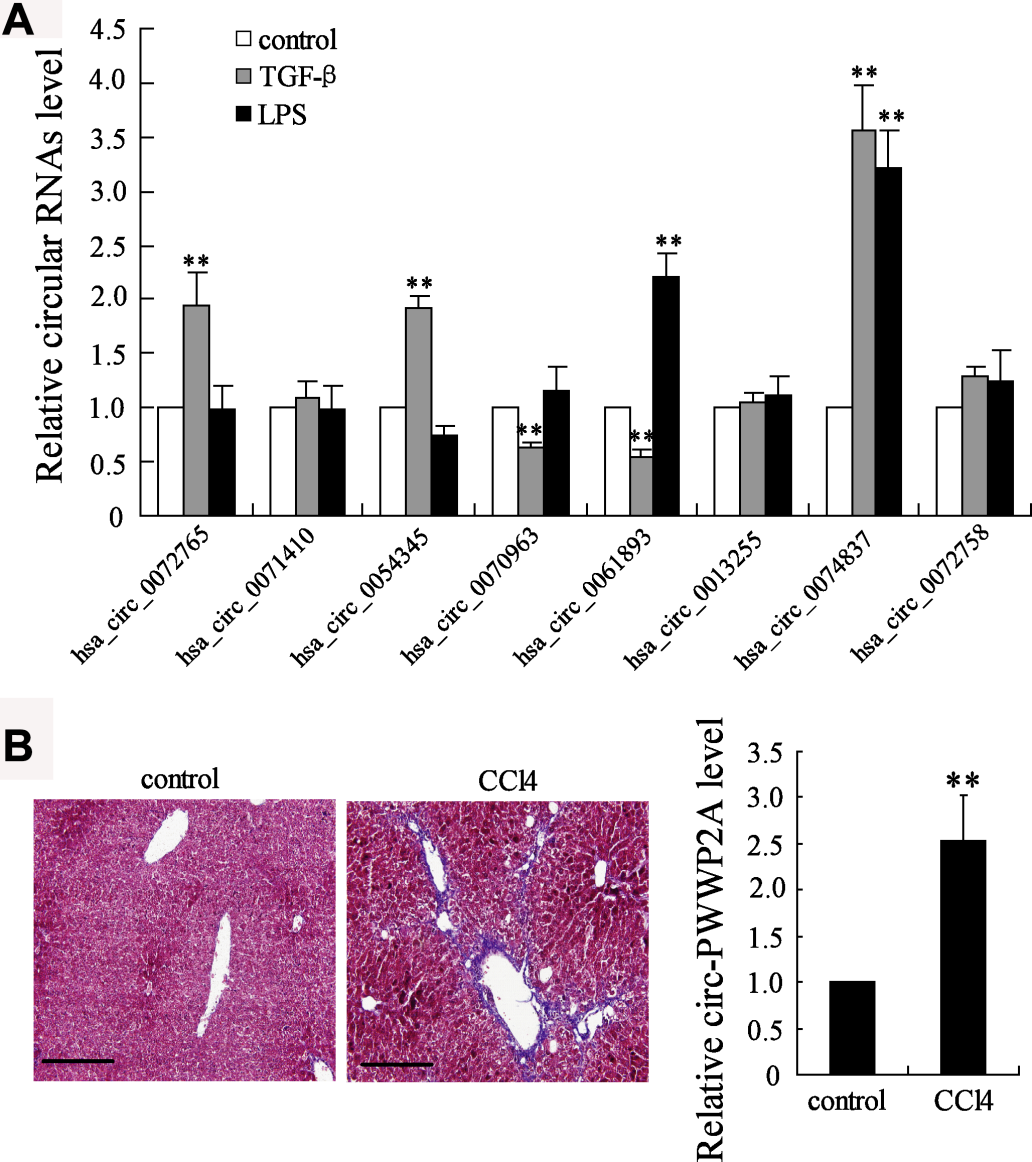
**Screening of circRNAs.** (**A**) Human HSC cell line LX-2 was activated by TGF-β (5 ng/ml) or LPS (5 μg/ml). After 24 hours, the expressions of several circRNAs, including hsa_circ_0072765, hsa_circ_0071410, hsa_circ_0054345, hsa_circ_0070963, hsa_circ_0061893, hsa_circ_0013255, hsa_circ_0074837 (the circ-PWWP2A in human genome), and hsa_circ_0072758, were detected in activated HSCs using qRT-PCR. (**B**) The fibrosis mouse model was established by i.p. injection of olive oil-dissolved CCl_4_ (0.3 ml/kg) thrice a week for 6 weeks. Liver tissues from the control group (n=7) and the CCl_4_ group (n=7) were collected for HE staining and the expression of mmu_circ_0000254 (the circ-PWWP2A in mouse genome) was detected using qRT-PCR. Magnification is 400X. Scale bar represents 200 μM. **p<0.01 vs control.

CircRNAs have been reported to take part in the activation of HSCs, the main fibrogenic cell type in hepatic fibrosis [19]. The different expressions of several circRNAs, including hsa_circ_0072765, hsa_circ_ 0071410, hsa_circ_0054345, hsa_circ_0070963, hsa_circ_0061893, hsa_circ_0013255, hsa_circ_0074837, and hsa_circ_0072758, were compared in TGF-β- or LPS-activated HSCs. The simultaneous enhancement of the expression of hsa_circ_0074837 (also named as circ-PWWP2A in human genome) in both TGF-β- and LPS-activated HSCs suggested it may play a critical role in HSC activation (Figure 1A). Then, its expression in CCl_4_-induced fibrosis mouse model was further detected, and the expression of mmu_circ_0000254 (the circ-PWWP2A in mouse genome) was markedly higher in fibrotic liver tissues than that in normal liver tissues (Figure 1B). Thus, circ-PWWP2A was chosen for further investigations against hepatic fibrogenesis.

### Circ-PWWP2A positively regulates the activation and proliferation of HSCs

**Figure 2:**
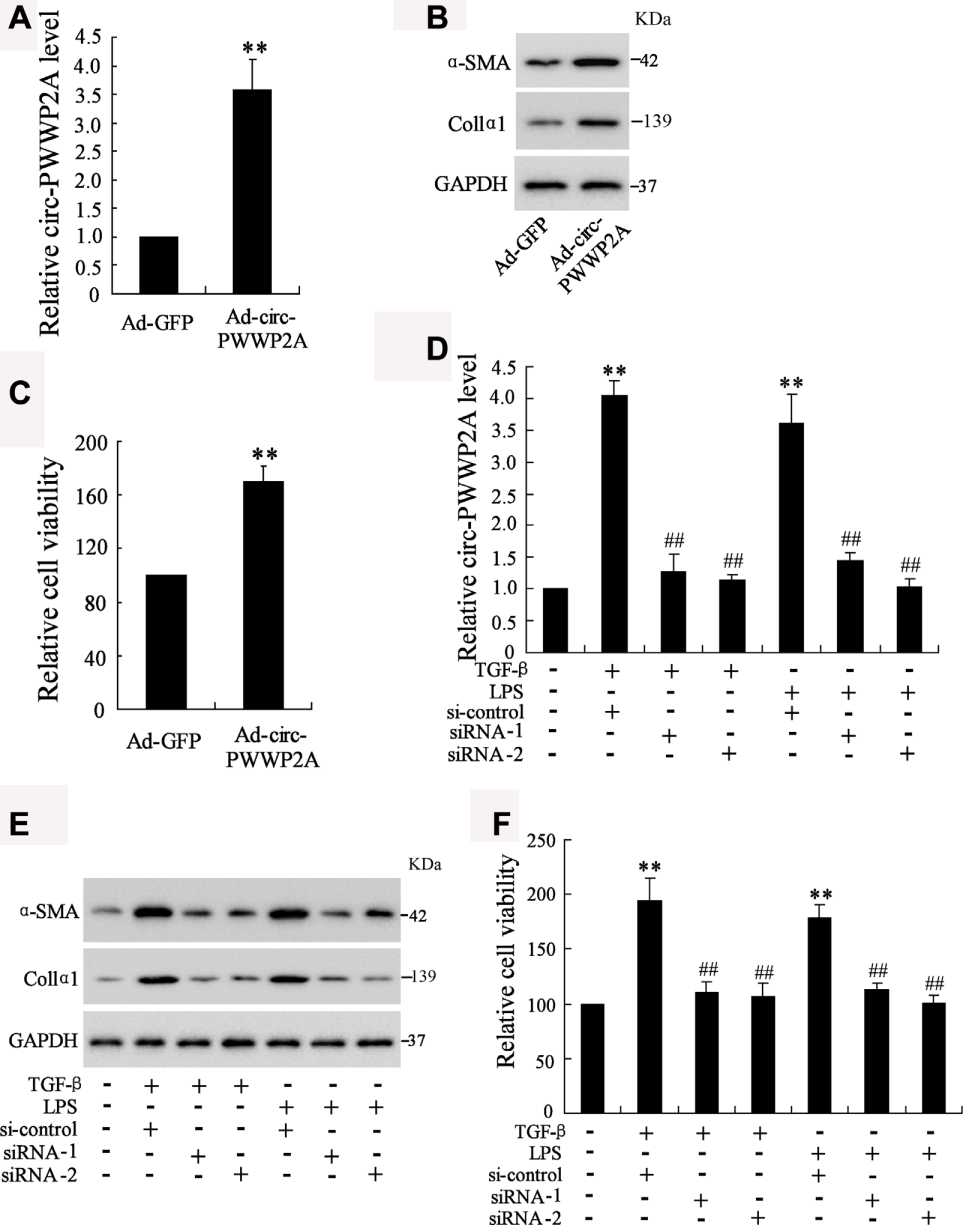
**Effect of circ-PWWP2A on activation and proliferation of HSCs.** (**A**) Overexpressing circ-PWWP2A was done by transfecting Ad-circ-PWWP2A into LX-2 cells. (**B**) Protein expression of α-SMA and Col1α1, both of which were activated HSC markers. (**C**) HSC proliferation was detected using CCK-8 assay. (**D**) Interfering circ-PWWP2A was done by transfecting siRNA of circ-PWWP2A (siRNA-1 and siRNA-2) in TGF-β- or LPS-activated LX-2 cells. (**E**) Protein expression of α-SMA and Col1α1. (**F**) Cell proliferation of activated-HSCs. **p<0.05 vs Ad-GFP or negative control; ##p<0.01 vs TGF-β+si-control or LPS+si-control. Ad-GFP, adenoviral vectors carrying green fluorescent protein genes that act as the control. CCK-8, cell counting kit-8. siRNA, small interfering RNA.

Given the upregulation of circ-PWWP2A in activated HSCs and fibrotic liver tissues, we further explored whether circ-PWWP2A regulates the activation and proliferation of HSCs. The result showed that overexpressing circ-PWWP2A using Ad-circ-PWWP2A transfection in LX-2 cells (Figure 2A) increased protein expression of α-SMA and Col1α1, both of which were activated HSC markers (Figure 2B), and it also promoted HSC proliferation (Figure 2C). Interfering circ-PWWP2A using siRNA of circ-PWWP2A in TGF-β- or LPS-activated LX-2 cells (Figure 2D) reduced protein expression of α-SMA and Col1α1 (Figure 2E), and it inhibited the proliferation of activated-HSCs (Figure 2F). These findings indicated that circ-PWWP2A positively regulates the activation and proliferation of HSCs.

### Circ-PWWP2A interacts with miR-203 and miR-223

**Figure 3:**
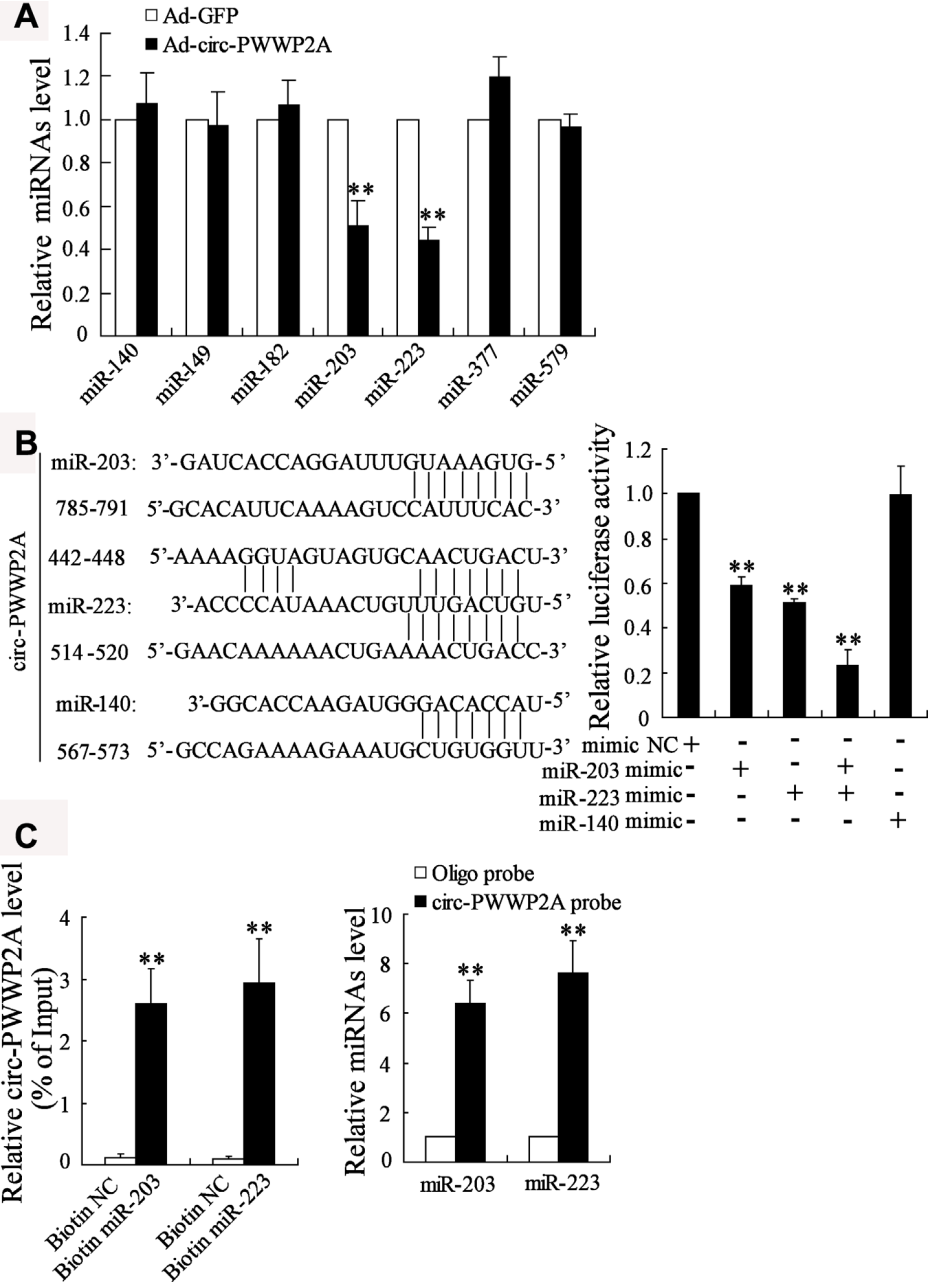
**Interaction between circ-PWWP2A with miR-203 and miR-223.** (**A**) The expression of several miRNAs which were predicted to bind with circ-PWWP2A by DIANA after overexpressing circ-PWWP2A in LX-2 cells. (B) After mutating the predicting sequences in circ-PWWP2A, the luciferase activity was detected using luciferase reporter assay. (C) The interaction between circ-PWWP2A with miR-203 and miR-223 was confirmed using agarose beads pull-down RNA-RNA interaction assay. **p<0.05 vs Ad-GFP. Ad-GFP, adenoviral vectors carrying green fluorescent protein genes that act as the control. NC, negative control.

Previous studies revealed that circRNAs exert their regulatory functions partly through acting as miRNA sponges [20]. To elucidate the miRNAs that could interact with circ-PWWP2A, we compared the different expressions of several miRNAs which were predicted to bind with circ-PWWP2A using the bioinformatics software DIANA. After overexpressing circ-PWWP2A in LX-2 cells, miR-203 and miR-223 appeared a significant downregulation, while the expression of miR-140, miR-149, miR-182, miR-377, and miR-579 was not much changed (Figure 3A). Then we mutated three sequences in circ-PWWP2A, which was predicted to bind with miR-203, miR-223, and miR-140. The result of luciferase reporter assay indicated that the co-transfection of miR-203 mimic and/or miR-223 mimic markedly reduced the luciferase activity, whereas the co-transfection of miR-140 mimic did not significantly change the luciferase activity (Figure 3B), suggesting miR-223 and miR-203 may be regulated by circ-PWWP2A. Such interaction was confirmed using pull-down assay as circ-PWWP2A was accumulated by miR-203 and miR-223 probes and miR-203/miR-223 was accumulated by circ-PWWP2A probe as well (Figure 3C).

### Circ-PWWP2A regulates Fstl1 and TLR4 via sponging miR-203 and miR-223, respectively

**Figure 4:**
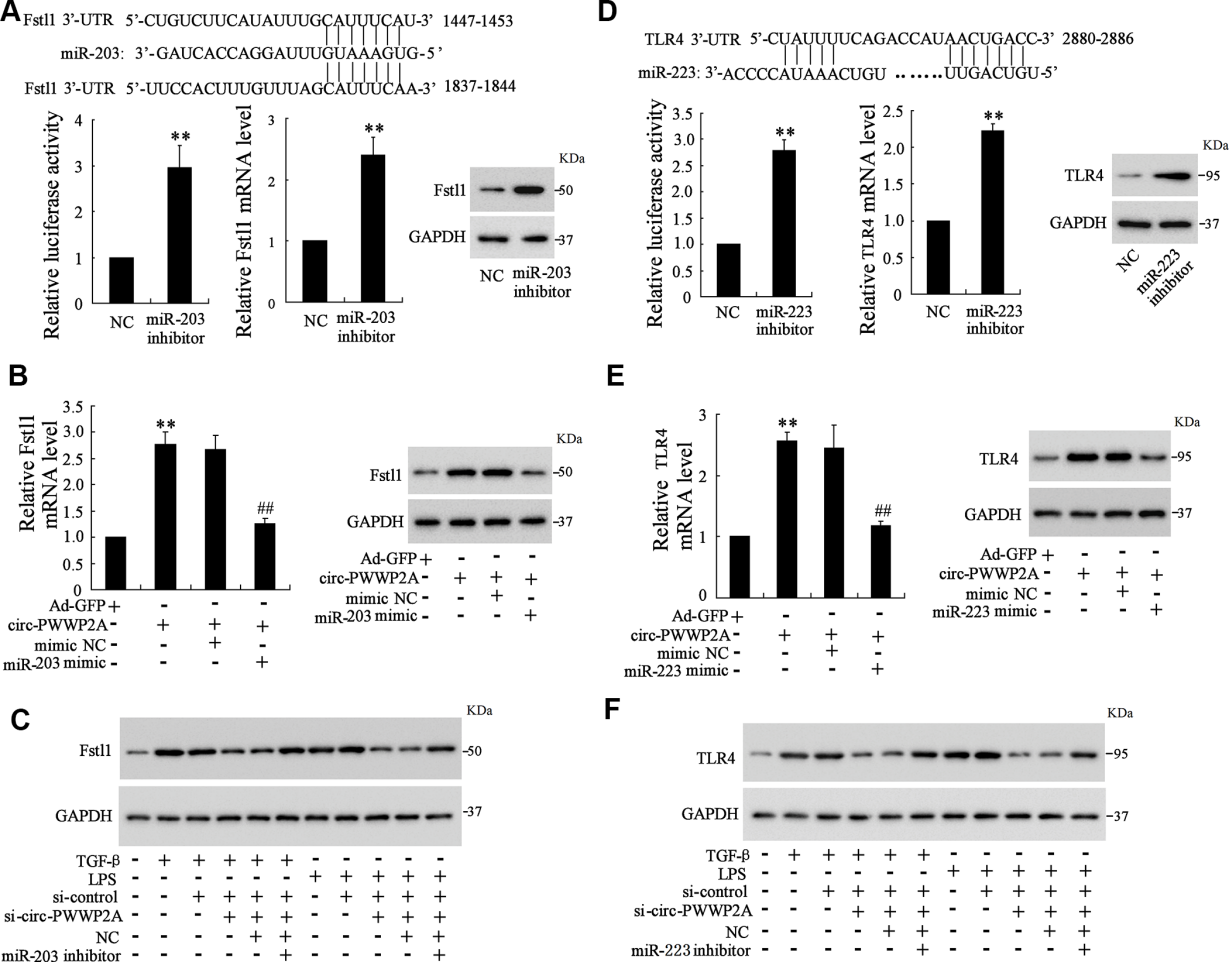
**Circ-PWWP2A regulates Fstl1 and TLR4 via sponging miR-203 and miR-223, respectively.** (**A**) The luciferase reporter vector carrying mutant Fstl1 3’-UTR was transfected into 293T cells along with miR-203 inhibitor, and the luciferase activity, mRNA and protein expressions of Fstl1 were detected. (**B**) LX-2 cells were transfected with Ad-circ-PWWP2A or co-transfected with miR-203 mimic. The mRNA and protein expressions of Fstl1 were detected. (**C**) LX-2 cells were transfected with si-circ-PWWP2A or co-transfected with miR-203 inhibitor followed by TGF-β- or LPS-activation. The protein expression of Fstl1 was detected. (**D**) The luciferase reporter vector carrying mutant TLR4 3’-UTR was transfected into 293T cells along with miR-223 inhibitor, and the luciferase activity, mRNA and protein expressions of TLR4 were detected. (**E**) LX-2 cells were transfected with Ad-circ-PWWP2A or co-transfected with miR-223 mimic. The mRNA and protein expressions of TLR4 were detected. (**F**) LX-2 cells were transfected with si-circ-PWWP2A or co-transfected with miR-223 inhibitor followed by TGF-β- or LPS-activation. The protein expression of TLR4 was detected. **p<0.01 vs NC or Ad-GFP; ##p<0.01 vs Ad-circ-PWWP2A+mimic NC. Ad-GFP, adenoviral vectors carrying green fluorescent protein genes that act as the control. NC, negative control. si-circ-PWWP2A, small interfering RNA of circ-PWWP2A.

As the interaction between circ-PWWP2A with miR-203 and miR-223 was elucidated, the targets of miR-203 and miR-223 remained unclear. Using the online predicting software Targetscan, we found the potential targets of miR-203 and miR-223 which were Fstl1 and TLR4, respectively. The luciferase reporter vector carrying mutant Fstl1 3’-UTR was transfected into 293T cells along with miR-203 inhibitor, and the luciferase activity was enhanced by such transfection. Meanwhile, miR-203 inhibition increased both mRNA and protein level of Fstl1 (Figure 4A). In LX-2 cells, simultaneously overexpressing circ-PWWP2A and miR-203 decreased the enhancement of mRNA and protein expression of Fstl1 raised by overexpressing circ-PWWP2A alone (Figure 4B). In TGF-β- or LPS-activated LX-2 cells, simultaneously interfering circ-PWWP2A and miR-203 restored the defect of Fstl1 protein expression caused by interfering circ-PWWP2A alone (Figure 4C). These data indicated that circ-PWWP2A regulates Fstl1 via sponging miR-203, and the similar relationship of circ-PWWP2A/miR-223/TLR4 was confirmed in Figure 4D–4F.

### Circ-PWWP2A promotes activation and proliferation of HSCs via regulating miR-203 and miR-223

**Figure 5:**
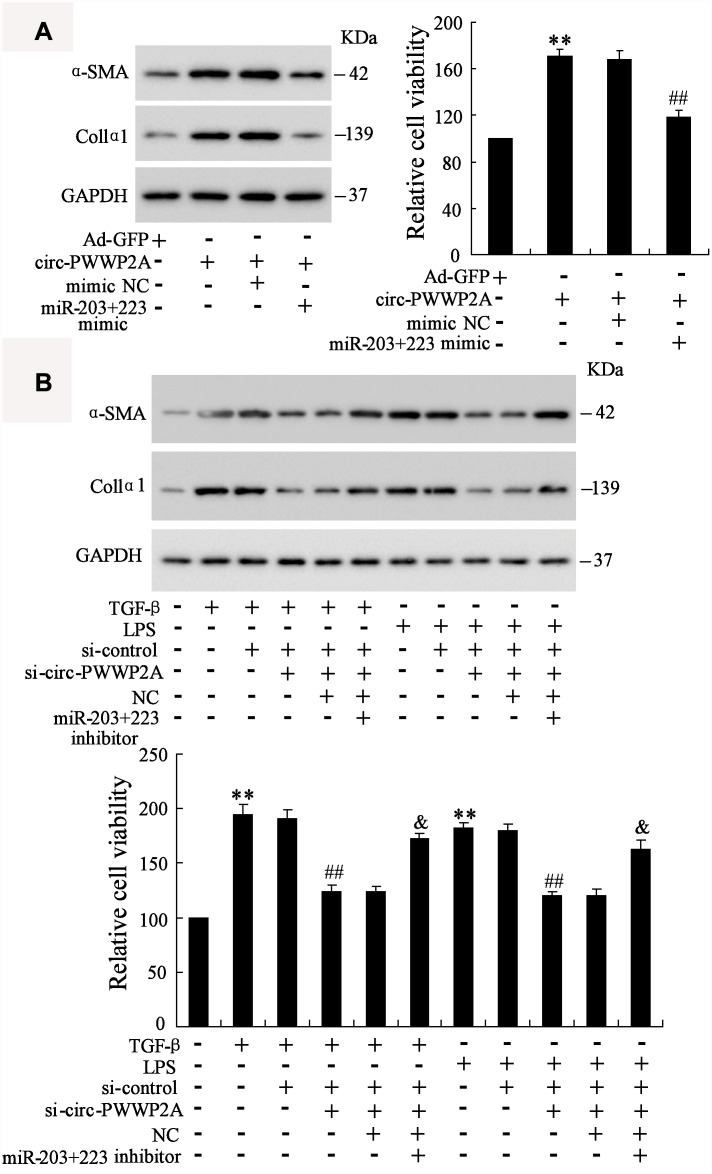
**Circ-PWWP2A promotes activation and proliferation of HSCs via regulating miR-203 and miR-223.** (**A**) LX-2 cells were transfected with Ad-circ-PWWP2A or co-transfected with miR-203+223 mimic. The protein expression of α-SMA and Col1α1 and cell viability were detected using western blot analysis and CCK-8 assay, respectively. (**B**) LX-2 cells were transfected with si-circ-PWWP2A or co-transfected with miR-203+223 inhibitor followed by TGF-β- or LPS-activation. The protein expression of α-SMA and Col1α1 and cell viability were detected. **p<0.01 vs Ad-GFP or negative control; ##p<0.01 vs Ad-circ-PWWP2A+mimic NC, TGF-β+si-control, or LPS+si-control; &p<0.05 vs TGF-β+si-control+si-circ-PWWP2A+NC or LPS+si-control+si-circ-PWWP2A+NC. Ad-GFP, adenoviral vectors carrying green fluorescent protein genes that act as the control. CCK-8, cell counting kit-8. si-circ-PWWP2A, small interfering RNA of circ-PWWP2A. NC, negative control.

In LX-2 cells, overexpressing circ-PWWP2A increased the protein expression of α-SMA and Col1α1 and promoted HSC proliferation, whereas the co-transfection with miR-203+223 mimic negated such response (Figure 5A). In TGF-β- or LPS-activated LX-2 cells, interfering circ-PWWP2A reduced the protein expression of α-SMA and Col1α1 and inhibited HSC proliferation, whereas the co-transfection with miR-203+223 inhibitor negated such response (Figure 5B). These data suggested that circ-PWWP2A promotes activation and proliferation of HSCs via regulating miR-203 and miR-223.

### Downregulating circ-PWWP2A alleviates hepatic fibrosis in vivo

**Figure 6:**
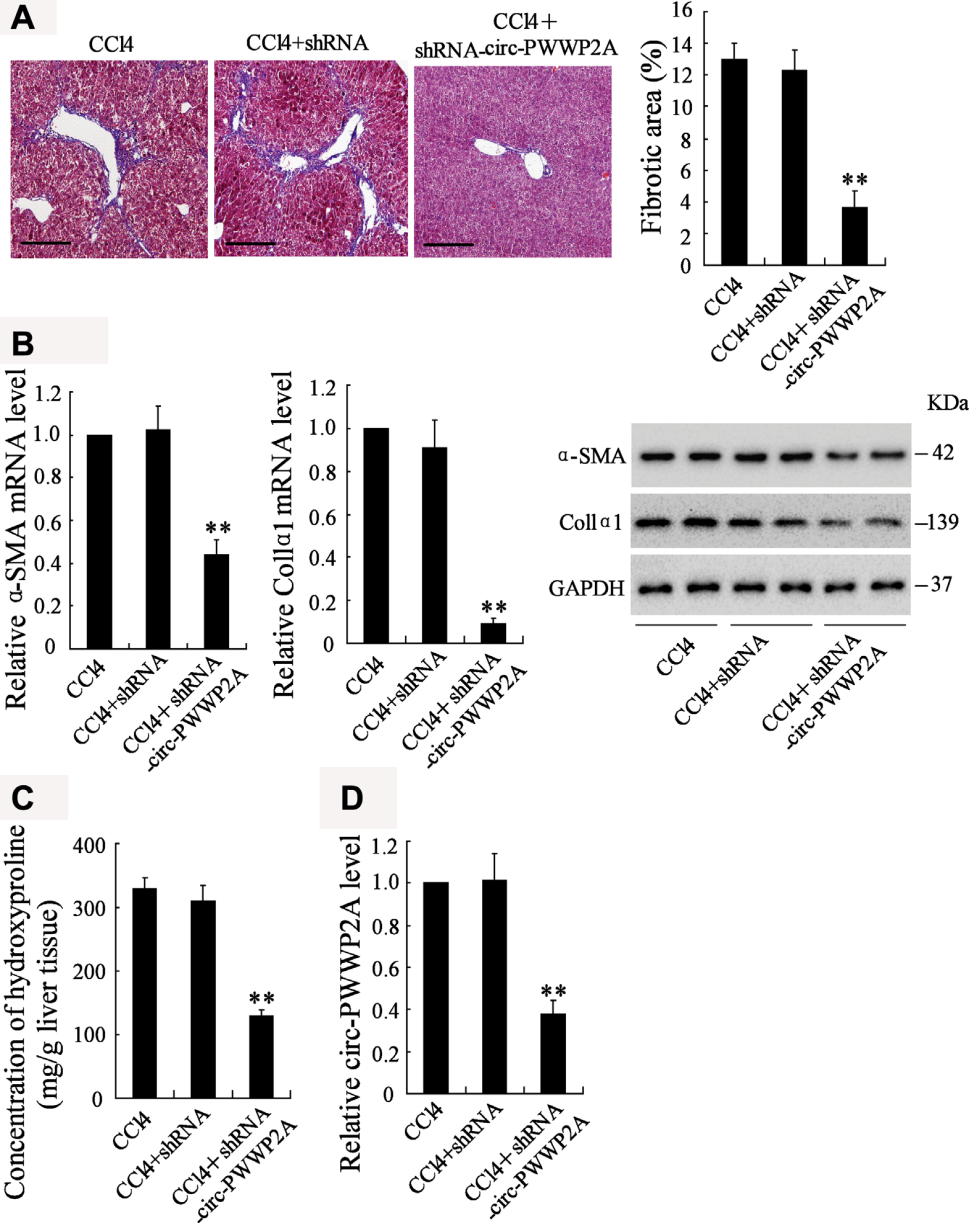
**Downregulating circ-PWWP2A alleviates hepatic fibrosis in vivo.** The adeno-associated virus carrying shRNA of circ-PWWP2A was injected into the CCl_4_-induced fibrosis mice at the third week of CCl_4_ injection. (**A**) Liver tissues from the CCl_4_ group (n=7), the CCl_4_+shRNA group (n=7), and the CCl_4_+shRNA-circ-PWWP2A group (n=7) were collected for HE staining, and the percentage of fibrotic area was calculated. Magnification is 400X. Scale bar represents 200 μM. (**B**) The mRNA and protein expressions of α-SMA and Col1α1. (**C**) The concentration of hydroxyproline (**D**) The expression of circ-PWWP2A. **p<0.01 vs CCl_4_+shRNA. shRNA, short hairpin RNA.

To investigate whether downregulating circ-PWWP2A alleviates hepatic fibrosis, shRNA of circ-PWWP2A was injected into the CCl_4_-induced fibrotic mice, and the therapeutic effects were observed. Before the injection, fatty degeneration and necrosis were clear in HE staining specimens of CCl_4_-induced liver tissues, while few necrotic cells and little fibrosis was shown after downregulating circ-PWWP2A. The percentage of fibrotic area also appeared a significant reduction after the injection (Figure 6A). In liver tissues, both the mRNA and protein expressions of α-SMA and Col1α1 were reduced (Figure 6B), and the concentration of hydroxyproline was reduced as well (Figure 6C). Taken together, these data suggested that downregulating circ-PWWP2A (Figure 6D) alleviated hepatic fibrosis in vivo.

.

## DISCUSSION

HSCs can be activated by both TGF-β and LPS, however, whether TGF-β and LPS have a common downstream reactor remains unclear. Our study demonstrated that circ-PWWP2A, which is upregulated in LPS and TGF-β induced HSCs, is the common downstream reactor for LPS and TGF-β. The pro-fibrotic function of circ-PWWP2A is mediated via sponging miR-203 and miR-223, thus subsequently increasing Fstl1 and TLR4.

CircRNA is produced by a non-canonical alternative splicing of pre-mRNAs, which is known as the back-splicing, where a downstream splice donor site is ligated to an upstream splice acceptor site reversely [21]. Usually, circRNAs are abundant in human cells, whose expression exceeds correlated linear mRNAs for 10 folds in some cases [22]. The circular structure of circRNAs makes them uneasy to be degraded, thus some highly expressed circRNAs play an important role in regulating pathogenic process, including fibrogenesis [11]. In hepatic fibrosis, circRNA_0071410 is indicated to be positively correlated with HSC activation and fibrogenesis [19], while circRNA MTO1 is considered to inhibit HSC activation and fibrogenesis [23]. In our study, we compared the expression of several circRNAs that were previously reported to be associated with fibrogenesis in activated HSCs, including hsa_circ_0072765, hsa_circ_0071410, hsa_circ_0054345, hsa_circ_0070963, hsa_circ_0061893, hsa_circ_0013255, hsa_circ_0074837, and hsa_ circ_0072758. The result indicated that hsa_circ_0074837 (also named as circ-PWWP2A in human genome) was significantly upregulated in both TGF-β- and LPS-activated HSCs and served as the common downstream reactor for LPS and TGF-β. Moreover, the expression of hsa_circ_0072765, hsa_circ_0054345, hsa_circ_ 0070963, and hsa_circ_0061893 was markedly changed by TGF-β or LPS stimulation. As these circRNAs were also reported to be dysregulated in irradiation-induced HSCs [19], their potential effects on liver fibrosis will be explored in our future studies.

CircRNAs are most involved in post-transcriptional regulation, which means circRNAs can act as a ceRNA by interacting with miRNAs and subsequently release target mRNAs from miRNAs, therefore increasing target mRNA expression [24]. Such ceRNA mechanism has been well-identified in long non-coding RNA/miRNA/mRNA regulatory system, while it is gradually accepted that such mechanism also occurs in circRNAs/miRNA/mRNA in recent years [25]. For instance, circRNA CDR1as, an antisense human circRNA derived from CDR1, is reported to suppress miR-7 and subsequently increase the expression of TGFBR2, the miR-7 target gene [18]. Apart from being a miRNA sponge, circRNAs are reported to directly bind with proteins. Du [26] and his colleagues demonstrated that circRNA Foxo3 arrests CDK2 function and blocks progression of cell cycle via directly binding with CDK2 and p21. In the current study, we compared the expressions of several miRNAs that were predicted to have potential interaction with circ-PWWP2A in HSCs, and demonstrated the direct binding between circ-PWWP2A with miR-203 and miR-223 using luciferase reporter assay and pull-down RNA interaction assay. In addition, the ceRNA mechanism of circ-PWWP2A/miR-203/Fstl1 and circ-PWWP2A/miR-223/TLR4 was confirmed in regulating HSC activation and proliferation. However, whether circ-PWWP2A also exerts its function via binding with proteins deserves further investigations.

Fstl1 (Follistatin-like protein 1), also named as TGF-β-stimulated clone 36 (TSC-36) or Follistatin related protein (FRP), can be upregulated by TGF-β stimulation in lung fibrosis [27]. In TGF-β-induced HSC, knockdown of Fstl1 effectively inhibited the TGF-β-induced HSC proliferation, α-SMA and type I collagen expression via suppressing TGF-β/Smads signaling pathway, suggesting the critical role of Fstl1 in HSC activation and pro-fibrogenesis [28]. TLR4, a molecule contributing to the progression of hepatic fibrosis, can be activated by LPS. Meanwhile, TLR4 activation induced by LPS sensitizes HSCs to TGF-β-induced signals via downregulating TGF-β pseudoreceptor in quiescent HSCs [29]. These data indicate a vital role of Fstl1 and TLR4 in TGF-β- and LPS-induced HSC activation. In our work, circ-PWWP2A is shown to be the mediator of Fstl1 and TLR4, promoting expressions of Fstl1 and TLR4 via reducing the inhibitory effect of miR-203 and miR-223 on mRNAs of Fstl1 and TLR4. The downregulation of circ-PWWP2A in fibrosis mouse model attenuates hepatic fibrogenesis, which applied the basis for developing therapeutic strategy in the future.

In conclusion, our findings indicated that circ-PWWP2A is the common downstream reactor for LPS and TGF-β and is upregulated in LPS and TGF-β induced HSCs, playing a critical role in HSC activation and proliferation. More effects of circ-PWWP2A on liver fibrogenesis deserve further investigations in the future.

## MATERIALS AND METHODS

### Cell line and cell activation

Human HSC line LX2 cells were cultured in the Dulbecco's modified Eagle's medium supplemented with 1% penicillin/streptomycin and 2% fetal bovine serum (FBS) [30]. HSC activation was performed by adding TGF-β (5 ng/ml) or LPS (5 μg/ml) to the medium. After 24-hour treatment, the cells were harvested for further experiments.

### Quantitative real-time PCR (qRT-PCR)

According to the manufacturer's instructions, total RNAs were extracted from LX-2 cells and liver tissues using Trizol reagent (Invitrogen, Grand Island, NY, USA). Real-time PCR was performed to quantify the RNA expression using an ABI ViiA 7 qPCR system (Applied Biosystems, Carlsbad, CA, USA) after reverse transcription, with a Powerup SYBR Green Master Mix (Applied Biosystems). Gene expressions were normalized against the U6 or GAPDH. Fold changes were calculated using the delta delta Ct method. The primers used in the study are described as follows: hsa_circ_0074837 (human): 5′-AAGACAGGACTTG AGAAAATGC-3′ (F), 5′-GGCATGGCTTC TGGTT TATC-3′ (R); mmu_circ_0000254 (mouse): 5′-CTGGA GAAAATTCGGAGTGG-3′ (F), 5′-GCGTCCGGTTT GTCCTTAT-3′ (R); hsa_circ_0072758: 5′-TGATC GGTTCATGCAGGTTG ATAC-3′ (F); 5′-TCCAGAT GTTTCCATTGGGCTTGG-3′ (R); hsa_circ_0072765: 5′-TTGCAGCAGGAGCTTTTTGC-3′ (F), 5′-GGCCA AAGTATGTTGCTCGAC-3′ (R); hsa_circ_0071410: 5′-AGGAAAACTCTGCAGAATGGACTG-3′ (F), 5′-CGTATGTTAACCTCG GCTGGC-3′ (R); hsa_circ_ 0054345: 5′-ATGCTGAAACCTACAGCAATCTCAT-3′ (F), 5′-GCATCAGCTCTTTGCCAGTCA-3′ (R); hsa_circ_0070963: 5′-TTCAACAACTGACCAAACA ACTTCA-3′ (F), 5′-CATCTCACCCACCTGTTTCTG G-3′ (R); hsa_circ_0061893: 5′-GGACCTCCTTCC CGTCTACAA-3′ (F), 5′-GGACAGAGTTCACCG CGT-3′ (R); hsa_circ_0013255: 5′-GAAATGCATAT TTGCCACAAAACCT-3′ (F), 5′-TGACACCAATATG CA GCCGT-3′ (R); GAPDH: 5′-CTGGGCTACACT GAGCACC-3′ (F), 5′-AAGTGGTCGTTGAGGGCA ATG-3′ (R).

### Western blot analysis

Cell extracts were obtained using RIPA buffer (pH 8.0, 50mM Tris, 0.1% SDS, 0.5% sodium deoxycholate, 1% Triton X-100, and 150mM NaCl) supplemented with complete EDTA-free protease inhibitor cocktail tablets (Roche, Basel, Switzerland) and phosphatase inhibitors (50 mM sodium vanadate, 10 mM 4-nitrophenylphosphate, and 20 mM b-glycerophosphate, Sigma Aldrich, St. Louis, MO, USA). Primary antibodies (all from Abcam, Cambridge, UK), including anti-α-SMA, anti-Col1α1, anti-Fstl1, anti-TLR4, and anti-GAPDH, were diluted with 3% BSA TBS-tween solution at different concentrations. The proteins were observed using ECL chemoluminescence (GE Amersham, Arlington Heights, IL).

### Hematoxylin-eosin (HE) staining

The liver tissue sections were fixed in 10% formalin, embedded in paraffin, cut into 4-μm-thick slides. The specimens were dewaxed 3 times with xylene for 5-10 min. After the alcohol dehydration, the specimens were stained with hematoxylin solution for about 4 minutes, and treated with 1% hydrochloric acid alcohol and 0.5% ammonia water until the nucleus turned blue, and then stained with eosin for 1 min. After being rinsed by alcohol, the specimens were again treated with xylene three times. The specimens were observed under a microscope and photographed.

### Cell viability

Cells were seeded in 96-well plates at a density of 2×10^3^ cells per well followed by the culture of 48 hours. Cell viability was determined using the Cell Counting Kit-8 (CCK-8) assay (Beyotime, Beijing, China), according to the manufacturer’s protocol. Absorbance was detected at the wavelength of 450 nm.

### Establishment of vectors

To over-express circ-PWWP2A, the genomic sequence for circ-PWWP2A with the 500 bp upstream and 500 bp downstream sequence was chemically synthesized by GeneChem (Shanghai, China). These sequences could allow the intervening exons of PWWP2A to circularize. They were cloned into adenoviral vectors thus the Ad-circ-PWWP2A was established. The adenoviral vectors carrying green fluorescent protein gene (Ad-GFP) were used as the negative control (NC). The vector preparations were purified by dialysis and tittered by qRT-PCRs. The circ-PWWP2A-interfering adeno-associated virus vector (shRNA-circ-PWWP2A) was established similarly.

### Cell transfection

For transfection, LX-2 cells were plated in 6-well plates at a density of 1×10^5^ cells per well and were cultured overnight. Cells were transiently transfected with mimics/inhibitors of miR-203 or miR-223 with a final concentration of 100 nM using Lipofectamine 2000 (Invitrogen). The mimics/inhibitors of miR-203 or miR-223 and their corresponding controls mimic negative control/negative control were bought from GeneChem. Cells were also transfected with Ad-circ-PWWP2A and its control Ad-GFF, or small interfering RNAs of circ-PWWP2A (siRNA-1 and siRNA-2) and their control (si-control) using Lipofectamine 2000 (Invitrogen) in accordance with manufacturers’ protocols. After 48 hours, cells were collected for further experiments.

### Luciferase reporter assay

The entire circ-PWWP2A sequence was inserted into pGL3 luciferase reporter to create a LUC-circ-PWWP2A vector. LX-2 cells were transiently co-transfected with miR-140 mimic, miR-203 mimic and/or miR-223 mimic with LUC-circ-PWWP2A vector. The luciferase activity was assayed according to the manufacturer's instructions. Results are presented as the relative luciferase activity of firefly to Renilla luminescence.

To explore the relationship between miR-203 and Fstl1, mutant or wild-type 3′-UTRs of Fstl1-containing putative complementary binding site were cloned into GV272-luciferase vector (Genechem). 293T cells were co-transfected with miR-203 inhibitor and mutant or wild-type 3′-UTR of Fstl1 vector. To explore the relationship between miR-223 and TLR4, mutant or wild-type 3′-UTRs of TLR4-containing putative complementary binding site were cloned into GV272-luciferase vector (Genechem). 293T cells were co-transfected with miR-223 inhibitor and mutant or wild-type 3′-UTR of Fstl1 vector. The luciferase activity was assayed according to the manufacturer's instructions. Results are presented as the relative luciferase activity of firefly to Renilla luminescence.

### RNA interaction assay

The interactions between circ-PWWP2A with miR-203 and circ-PWWP2A with miR-223 were determined using Agarose Beads Pull-Down RNA-RNA Interaction Assay [31]. LX-2 cells were transfected with biotin-labeled miR-203 or miR-223, and incubated with agarose beads for 20 min at 30 °C. Then the beads were washed 3 times with excess folding buffer followed by 1 min heating at 90 °C to release the RNA. The expression of circ-PWWP2A was detected using qRT-PCR. The circ-PWWP2A probe was also used to accumulating miR-203 and miR-223, and the expression of miR-203 and miR-223 was detected by qRT-PCR.

### Carbon tetrachloride- (CCl_4_-) induced fibrosis mouse model

A total of 35 Balb/c male mice, weighting from 18 to 25 g, with the age of 8 weeks were bought from the Laboratory Animal Center of Zhengzhou University. They were maintained in a 12-hour light/dark room at about 24 °C, and were free to get food and water. For the screening of circRNAs, the fibrosis mouse model was induced by CCl_4_ as previously described [32]. In brief, 14 mice were randomly divided into 2 groups, the control group (n=7) and the CCl_4_ group (n=7). The CCl_4_ solution (v/v, Sigma-Aldrich, St. Louis, MO, USA) was dissolved in olive oil with a final concentration of 5%. Then the mice in the CCl_4_ group were intraperitoneally injected with CCl_4_ at a dose of 0.3ml/kg 3 times a week for 6 weeks. The mice in the control group were intraperitoneally injected with the equal amount of olive oil alone. For the in vivo experiment, 21 CCl_4_-induced mice were randomly assigned into 3 groups, the CCl_4_ group (n=7), the CCl_4_+shRNA group (n=7), and the CCl_4_+shRNA-circ-PWWP2A group (n=7). After 2 weeks of CCl_4_ injection, mice in the CCl_4_+shRNA-circ-PWWP2A group were intravenously injected via the tail vein with the shRNA-circ-PWWP2A at a dose of 300 μl viruses/mouse (10^12^ genome copy particle/ml). Mice in the CCl_4_+shRNA group were injected with the negative control adeno-associated virus. After 6-week induction, mice were sacrificed and liver tissues were harvested for testing.

### Statistical analysis

Data were presented as mean ± standard deviation (SD). SPSS software (version 20.0; Chicago, USA) was used in analysis. The significance of difference between two groups was detected using t test. The comparisons between groups were done using one-way analysis of variance (ANOVA), followed by Tukey post hoc testing. We consider a p value less than 0.05 as statistically significant.

### Ethics approval

This study was approved by the Ethical Committee of The First Affiliated Hospital of Zhengzhou University. All animals-treatment operations were executed according to the Zhengzhou University Ethical Guidelines for Animal Experiment.
